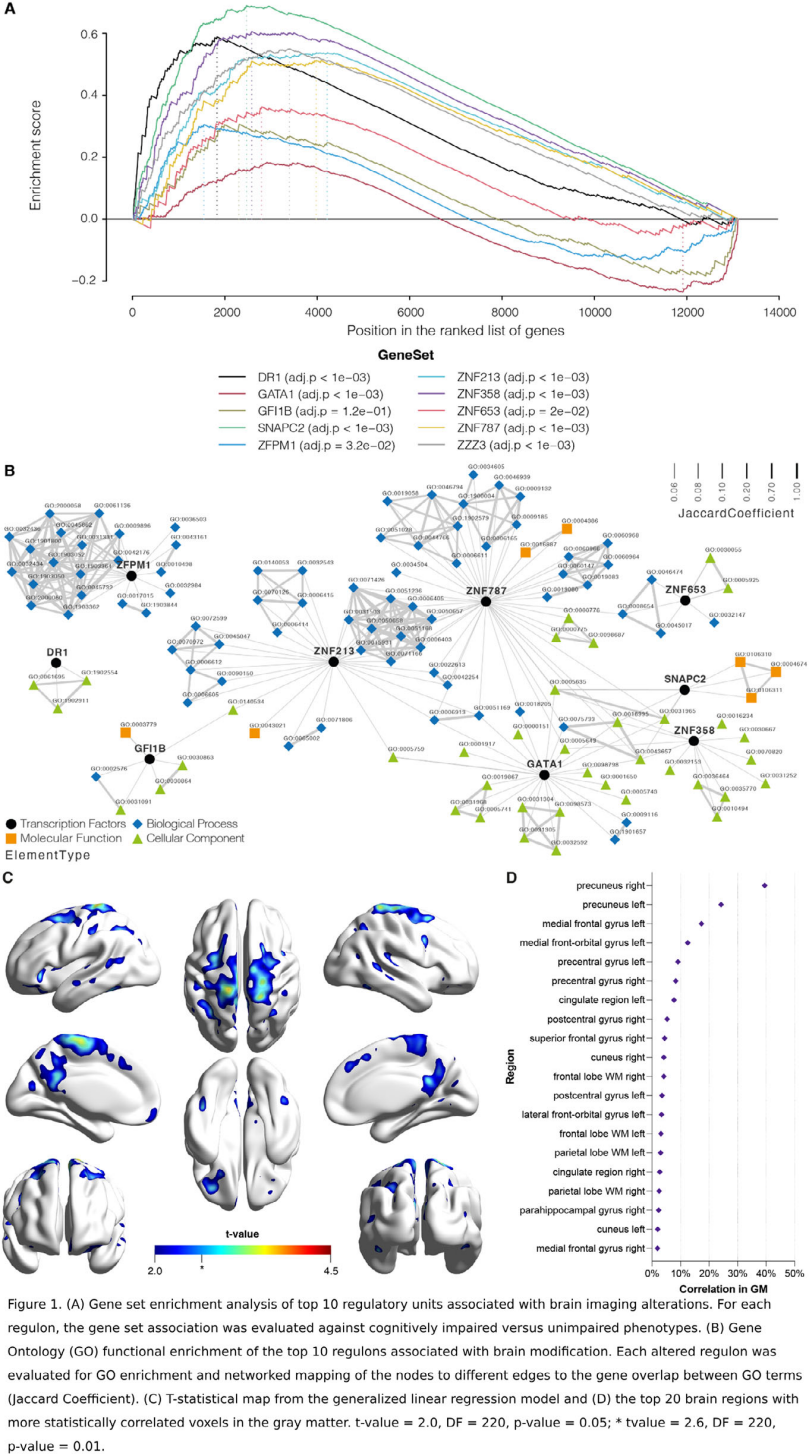# ZNF653 transcription factor activity is associated with FDG‐PET changes in AD brain‐vulnerable regions

**DOI:** 10.1002/alz.084700

**Published:** 2025-01-03

**Authors:** Marco De Bastiani, Guilherme Povala, Bruna Bellaver, Pamela C.L. Ferreira, João Pedro Ferrari‐Souza, Pedro Rosa‐Neto, Bruno Zatt, Tharick A. Pascoal, Eduardo R. Zimmer

**Affiliations:** ^1^ UFRGS, Porto Alegre, PA Brazil; ^2^ University of Pittsburgh, Pittsburgh, PA USA; ^3^ Universidade Federal do Rio Grande do Sul, Porto Alegre, Rio Grande do Sul Brazil; ^4^ McGill University, Montreal, QC Canada; ^5^ Universidade Federal de Pelotas, Pelotas Brazil

## Abstract

**Background:**

Positron emission tomography (PET) imaging greatly impacted Alzheimer’s disease (AD) research and diagnosis. which makes predicting PET brain imaging alterations using blood data is of high interest. Additionally, integrating PET and omics data can provide new insights into AD pathophysiology. Here, we implemented a module‐based framework combining blood transcriptomics with PET to search transcription factors (TFs) activities associated with brain metabolic changes in AD. We hypothesized that integrating omics and PET data will help advance our understanding of AD neurobiology and may reveal relevant new peripheral biomarkers.

**Methods:**

[^18^F]Fluorodeoxyglucose ([^18^F]FDG)‐PET imaging and transcriptomics data were acquired from the Alzheimer’s Disease Neuroimaging Initiative (ADNI). Blood microarray gene expression from ADNI, GSE63063 and GSE97760 (https://www.ncbi.nlm.nih.gov/geo/) datasets were submitted to differential expression (DE) analysis. Regulatory units (regulons) of TFs and their predicted target genes were reconstructed using the ARACNe method. Altered regulons in AD submitted gene set variation analysis to infer their TF activity prior to neuroimaging integration with [^18^F]FDG‐PET images using voxel‐wise eneralized linear regression (GLR) models adjusted for age, gender, years of education, and APOEε4 (RMINC package).

**Results:**

Sixty‐one regulatory units were significantly enriched with altered genes in at least ⅔ of the datasets explored, and 12 were altered in all three (Figure A‐B). The voxel‐wise correlation between [^18^F]FDG‐PET and regulons resulted in t‐statistical maps, where uncorrected t‐value > 2.0 was used as the threshol. We observed that ZNF653 has a positive correlation with [^18^F]FDG‐PET in the precuneus (24.24% left, 39.51% right), medial frontal gyrus (17.26% left), medial frontal‐orbital gyrus (12.50% left) and precentral gyrus (9.08% left, 8.28% right). Interestingly, the ZNF653 regulatory unit is composed majoritarily by genes related to energetic metabolism and protein kinase activity (Figure 1C‐D).

**Conclusion:**

We identified the activity of the ZNF653 regulatory unit associated with [18 F]FDG‐PET metabolism in the brain of AD individuals. Furthermore, ZNF653 activity could be modulating metabolic and protein kinase activity‐related genes, highlighting a potential role of this TF in AD.